# Using Twitter Data for the Study of Language Change in Low-Resource Languages. A Panel Study of Relative Pronouns in Frisian

**DOI:** 10.3389/frai.2021.644554

**Published:** 2021-04-15

**Authors:** Jelske Dijkstra, Wilbert Heeringa, Lysbeth Jongbloed-Faber, Hans Van de Velde

**Affiliations:** ^1^Fryske Akademy, Leeuwarden, Netherlands; ^2^Mercator European Research Centre on Multilingualism and Language Learning, Leeuwarden, Netherlands; ^3^Faculty of Arts and Social Sciences, Maastricht University, Maastricht, Netherlands; ^4^Department of Languages, Literature and Communication, Utrecht University, Utrecht, Netherlands

**Keywords:** CMC, Frisian, relative pronoun, t-deletion, panel study, frequency, methodology

## Abstract

This paper investigates the usability of Twitter as a resource for the study of language change in progress in low-resource languages. It is a panel study of a vigorous change in progress, the loss of final t in four relative pronouns (*dy't, dêr't, wêr't, wa't*) in Frisian, a language spoken by ± 450,000 speakers in the north-west of the Netherlands. This paper deals with the issues encountered in retrieving and analyzing tweets in low-resource languages, in the analysis of low-frequency variables, and in gathering background information on Twitterers. In this panel study we were able to identify and track 159 individual Twitterers, whose Frisian (and Dutch) tweets posted in the era 2010–2019 were collected. Nevertheless, a solid analysis of the sociolinguistic factors in this language change in progress was hampered by unequal age distributions among the Twitterers, the fact that the youngest birth cohorts have given up Twitter almost completely after 2014 and that the variables have a low frequency and are unequally spread over Twitterers.

## Introduction

Since the spread of the Internet and social media, language use on the Internet has drawn the attention of scholars in linguistics (Herring, 1996; Crystal, 2001) and communication (Thurlow et al., 2004). It resulted in numerous studies on various topics within the domain of computer-mediated communication (CMC) such as: bilingual practices (Cunliffe et al., 2013; Androutsopoulos, 2014; Jongbloed-Faber et al., 2016, 2017; Reershemius, 2017; Cutler and Røyneland, 2018), discourse strategies (Herring, 2001; Herring and Paolillo, 2006; Baron, 2010; Androutsopoulos, 2014), and spelling skills (Plester et al., 2008; Stæhr, 2015). Scholars studying language change in progress got interested in CMC too, although the number of studies remains relatively low. For example, Eisenstein (2013, 2015) showed that in American English tweets t/d-deletion depends on its phonological context, but the effect is less outspoken than in speech. Grieve et al. (2019) compared regional lexical variation in British English between Twitter data and traditional survey data. In both resources similar lexical patterns were identified, but some regional patterns showed up more clearly in Twitter than in survey data. Vandekerckhove (2006), Vandekerckhove and Nobels (2010), De Decker (2014), Grondelaers et al. (2017) and Verheijen (2017) successfully used CMC corpora to study language variation and change in Dutch, a medium-sized language and the dominant language in the written domain in the Frisian language area, the geographical focus of our study (see below).

The scarceness of variation research in CMC is partly due to predominantly anonymous contributions in CMC. Consequently, information about the writer's demographic background such as gender, age, education, birth place, hometown and social class, is not directly available (Herring, 2001; Grieve et al., 2019), which hampers a variationist sociolinguistic analysis. Participants' gender and age can often be deducted from screen names or profile descriptions and pictures, but it is time consuming to search for this information. Although computer models have been built to automatically predict age and gender for large and medium-sized languages as English and Dutch (Nguyen et al., 2013) or for multilingual data (Wang et al., 2019), such models are not available for smaller languages or dialects. Furthermore, the demographic background of users of specific social media platforms differs from the offline population's background. E.g., Twitterers in the UK and the US are more likely to be younger, better educated, students or employed, single and wealthier compared to the other Internet users and the offline population (Blank, 2016). This creates a bias in research results based on Twitter data. Blank therefore discourages the use of Twitter data in social sciences.

The stylistic characteristics of language practices has been a central topic from the early start of CMC research. The use of non-standard spelling and the presence of spoken language features show up as core linguistic features of CMC. Consequently, CMC language does not correspond to traditional written communication, in which writers generally conform to spelling rules and discard spoken features. Nevertheless, language in CMC cannot be gathered completely under the concept of oral communication either, because many contextual cues that are available in spoken language, e.g., intonation and facial expression, are not possible in CMC. In other words, language practices in CMC hold somewhere between written and oral communication (Androutsopoulos, 2014). That said, one may wonder whether CMC language is an appropriate source for language variation research. Stæhr (2015) argued that precisely the presence of colloquial features in CMC language pleads for inclusion of language from digital media in the study of language variation. De Decker et al. (2016) concluded that CMC language such as chatspeak can be a useful source to study language variation, if the variables are well-chosen and analyzed, according to the standards in variationist sociolinguistic research. Additionally, Grondelaers et al. (2017) demonstrated that, despite its limitation in number of characters, tweets are a rich resource to study morphosyntactic variation as well.

Bleaman (2020) pointed out that most of the sociolinguistic studies of social media focus on a handful of languages and that minority language are neglected, due to scarcity of data and the lack of computational tools to collect and analyze data of these languages and language varieties. He observes that CMC studies of low-resource varieties have mainly focused on macro-level analyses, and not on the analysis of linguistic variables at the micro-level. Bleaman was able to trace and analyze a syntactic change in progress in a real time corpus (2012–2019) of discussion forums written in Hassidic Yiddish, a low-resource language.

In this paper, we will further explore the possibilities that social media offer for the study of language variation and change in low-resource languages. We first give a brief introduction to Frisian and the language situation in Fryslân. More detailed information can be found in Munske et al. (2001) and Jonkman and Versloot (2018). Frisian is an autochtonous minority language spoken in the province of Fryslân (the Netherlands), where it is recognized as an official language, in addition to Dutch. Both are closely-related West-Germanic languages, but mutual intelligible. Eighty-nine percent of the inhabitants in Fryslân report to understand Frisian, whereas 69% is able to speak it (very) well. Sixty-one percent of the population, about 400,000 people, is a native speaker of Frisian (Klinkenberg et al., 2018). All speakers of Frisian speak Dutch too, and most of them write mainly in Dutch.

Although Frisian has an official written standard, most of the native speakers do not read or write Frisian. In the most recent language survey, 18% of the Frisians indicate they can write it well or very well (Klinkenberg et al., 2018), but it should be noted that this increase (doubled in comparison with surveys in the 1980s and 1990s), is linked to an increasing use of Frisian in social media. Up to today, the two regional daily newspapers are written in Dutch and only occasionally use Frisian in for example quotes (Gorter, 2001). Consequently, for most Frisians writing and reading is not an everyday activity. However, social media have changed this. Jongbloed-Faber et al. (2016) showed that 87% of Frisian-speaking teenagers use Frisian on social media to some extent. On Twitter, 29% of the Frisian-speaking adolescents indicated they use Frisian often or all the time in addressed and 24% in general tweets. On WhatsApp and in chat messages on Facebook the proportion of teenagers using Frisian is even higher. While writing skills appear the most important predictive variable for the adults' use of Frisian on social media (Jongbloed-Faber, 2015), the language use with peers and attitudes are more important than writing skills among adolescents (Jongbloed-Faber et al., 2016).

The change under investigation is the substitution of t-full relative pronouns, i.e., *dy't* ‘who/that’, *dêr't* ‘where’, *wêr't* ‘where,’ and *wa't* ‘who(m)’, by their t-less counterparts, i.e., *dy, dêr, wêr*, and *wa* respectively. This change in progress has been found in an earlier real-time study on this substitution in scripted and unscripted broadcast speech (Dijkstra et al., 2017, 2018, 2019), see Section Method for an overview of the results. In this study we attempt to get more insight in this vigorous change in progress, by analyzing a large data set. The variable is sensitive to normative grammatical rules: reference grammars prescribe the use of t-full relative pronouns (Popkema, 2018, pp. 175–177).

For several reasons we opted for Twitter as a source for our study:

- More monitoring of the writing process in comparison with WhatsApp messages (Verheijen, 2018).
- The length of the messages allows for the occurrence of more complex structures, including relative clauses (needed for our linguistic variable).
- Lack of other written media: there are almost no popular blogs written in Frisian.
- The medium is frequently used by a wide range of individuals and non-professional writers.
- As a public medium the data should be easily accessible for research.
- It exists long enough to cover a period of 10 years, a minimum needed to study language change in progress.
- Frisian teenagers reported to use Frisian frequently.
- Existence of a dataset that could help in the collection of a new dataset and enabling us to follow individuals over time.
- Allowing to study the interaction of the factors time and age.

To sum up, the current study has three research aims:

1. Explore the issues in gathering a Twitter corpus of a low-resource language such as Frisian.
2. Get insight in the validity of Twitter data for the study of language change in progress in low-resource languages.
3. Refine existing sociolinguistic insights in a vigorous change in progress in Frisian relative pronouns.

### Real-Time Change in Frisian Relative Pronouns

The Frisian relative pronouns ending in'*t* are *dy't* ‘who/that’, *dêr't* ’where’, *wêr't* ’where’, and *wa't* ’who(m)’. Examples of these pronouns are shown in Table 1.

**Table 1:** Examples of the Frisian relative pronouns *dy't, dêr't, wêr't*, and *wa't*.

**Relative pronoun**	**Example**			
*dy't*	*de man*	*dy't*	*in boek*	*lêst*
	the man	who-REL	a book	read-3SG
	‘the man who reads a book’			
*dêr't*	*de stêd*	*dêr't*	*er no*	*wennet*
	the city	there-REL	he now	live-3SG
	‘the city he currently lives in’			
*wêr't*	*ik wit net*	*wêr't*	*de kaai*	*is*
	I know-1SG not	where-REL	the key	be-3SG
	‘I do not know where the key is’			
*wa't*	*wa't*	*dat dien hat*	*is*	*in held*
	who-REL	that do-PP have-AUX	be-3SG	a hero
	‘whomever has done that, is a hero’			

The relative pronoun *dy't* ‘who/that’ is used with feminine, masculine and plural antecedents. There are two Frisian relative adverbs, namely *dêr't* and *wêr't* ‘where’. When the relative clause has an antecedent that is a location, then the adverb *dêr't* is used. In free relatives, *wêr't* is used. Due to the influence of Dutch, *dêr't* is often substituted by *wêr't* (De Haan, 2001; Dijkstra et al., 2018; Taalportaal|Relative pronouns, 2018) since the Dutch equivalent for both is *waar* ‘where’ which translates directly to *wêr* in Frisian (the Dutch translation of *dêr* is *daar* ‘there’). The relative pronoun *wa't* ‘who(m)’ is used in free relatives and refers to a person (Taalportaal|Relative pronouns, 2018).

The orthographic '*t* is found in other Frisian conjunctions as well. It marks the beginning of a subordinate sentence. The addition of '*t* to conjunctions is a relatively new phenomenon in Frisian. It is mentioned for the first time in *dy't* and *hwa't* [former spelling of *wa't*] as an option next to *dy* and *hwa* in a descriptive grammar of Frisian from 1889 (Van Blom, 1889 in Van der Woude, 1960).

**Figure 1:**
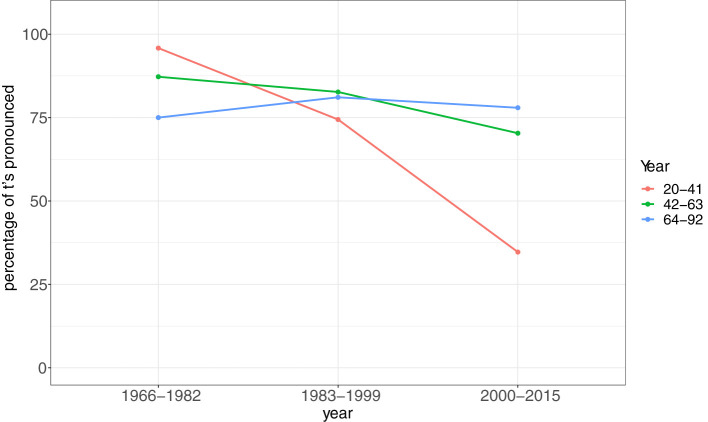
Percentages of t-full forms in (*dy't*), (*dêr't*), (*wêr't*), (*wa't*), interaction effect between age of all speakers and broadcasting year (*n* = 776, *N* = 266). Based on: Dijkstra et al. (2019, p. 95).

Recently three real time studies on the substitution of t-full Frisian relative pronouns by t-less forms were conducted using speech data from the radio archive of the regional broadcaster *Omrop Fryslân* (Broadcasting Corporation Fryslân) Dijkstra et al. (2017, 2018, 2019) looked at this substitution in Frisian relative pronouns in non-scripted speech (i.e., spontaneous speech), semi-scripted speech (i.e., mixture of pre-written catch words/phrases and spontaneous speech) and scripted speech (all text is read aloud) (see also Chignell, 2009). The general conclusion is that the t-full forms occurred more frequently in older broadcasts and in scripted speech. The substitutions by t-less forms showed a significant increase in recent broadcasts, especially in non-scripted speech. An interaction effect between age and broadcasting year was also found (see Figure 1). This figure clearly shows that in the older broadcasts the youngest age group lead the change toward more t-full forms (as mentioned in the descriptive grammar from 1889 for the first time). They produce more t-full forms compared to the oldest age group in the broadcasts from 1966 to 1982. This is in line with Brouwer (1959) who observed that the realization of (t) in Frisian conjunctions was increasing. In the most recent radio fragments (2000–2015), however, we see a reversal of the first observed language change: the speakers from the oldest age group use the t-full forms most frequently, whereas the youngest age group use the t-less forms the most. This latter pattern was already observed by Van der Meer (1991) and confirmed by these studies. Finally, it should be noted that the change is now in the quick or steep phase of the traditional S-curve pattern observed in language change.

In the current study we investigate whether we can refine our insights in this language change in progress on the basis of an analysis of Twitter data, covering a time span of 10 years. By following Twitterers over time (panel study) we want to get more insight in patterns of individual stability and change during the rapid spread of a change through the community, a core theoretical topic in variationist sociolinguistics (Wagner and Buchstaller, 2017). We expect to see a continuation of the reversal previously observed in speech data, thus that the Twitterers use more t-less forms over time in the 2010's. As previously stated, language practices on Twitter are situated between oral and written language use (Androutsopoulos, 2014) and spoken language features in tweets are more common amongst young Twitters (Androutsopoulos, 2006). This means that we expect that younger Twitterers use more t-less forms than older Twitterers.

## Method

### Collecting Twitter Data

Frisian tweets have been collected earlier in the Twidentity project using a language detector trained on identifying Frisian, Limburgish and Dutch tweets (Jongbloed-Faber et al., 2017). That Twitter data set comprises 76,757 predominantly Frisian tweets of 253 Twitter accounts posted in 2013 and 2014. This list of 253 Twitter accounts consists of 208 individual Twitter accounts (71,835 predominantly Frisian tweets) and 45 Twitter accounts that were owned by SMEs or organizations. Since we intended to conduct a panel study of language change in progress in Frisian relative pronouns, we decided to build a new corpus with all tweets of the abovementioned 208 individual Twitter accounts from the Twidentity project posted from January 1, 2010 until December 31, 2019, covering a time span of 10 years in real time.

### Method of Retrieving Tweets

Twitter's REST and stream Application Program Interfaces (API) are meant to be used for retrieving tweets. The R package rtweets provides several functions that use these APIs. The simplest is to use the function search_tweets, but this function only returns tweets from the past 6–9 days. Since we aim to retrieve tweets of a period of 10 years, we had rather to use the function search_fullarchive which uses Twitter's premium APIs. In order to be able to use this function, one needs to have a Twitter developer account which can be obtained for free. By using this function, we were able to retrieve 1,717 tweets after which we got the message: “Request exceeds account's current package request limits. Please upgrade your package and retry or contact Twitter about enterprise access.” Therefore, we applied for an enterprise API access at https://developer.twitter.com/en/enterprise-application twice, but never received any response.

Therefore, we used GetOldTweets3, a “Python 3 library and a corresponding command line utility” developed by Jefferson Henrique and forked by Dmitry Mottl (see: https://pypi.org/project/GetOldTweets3/). The authors describe the methodology they implemented as follows:

“Basically when you enter on a Twitter page a scroll loader starts. If you scroll down you start to get more and more tweets, all through calls to a JSON provider. After mimic we get the best advantage of Twitter Search on browsers, it can search the deepest oldest tweets.”

The tweets were collected late January and early February 2020. We wanted to limit our study to tweets sent by people who are still living in the province of Fryslân and are likely to interact in Frisian on a daily basis. This was operationalized by retrieving those accounts that were registered within a radius of 50 km from the village Grou, which is centrally located in the province. Due to closure of twitter accounts and emigration from Fryslân between 2013 and 2020, the number of Twitterers in our data set is 186.

We ran the GetOldTweets3 script in order to retrieve tweets between 1 January 2010 and 31 December 2019. When doing this a second time, we obtained a set of tweets that slightly differed from the first set. Therefore, we ran the script 14 times, and combined the 14 sets of tweets into one set. Tweets that appeared multiple times were kept only once. In this way, we maximized the number of tweets that we retrieved. In total we retrieved 698,369 tweets of 186 Twitterers.

### The Entire Twitter Data Set (698,369 Tweets of 186 Twitterers)

**Figure 2:**
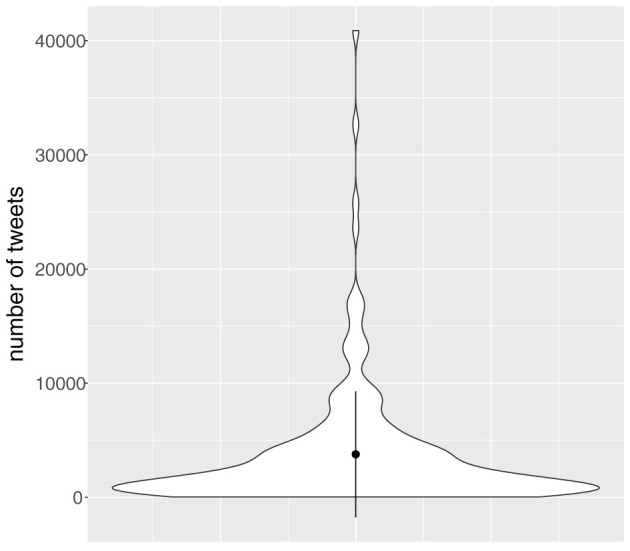
Violin plot showing the distribution of the number of tweets per Twitterer in the period from January 1, 2010 until December 31, 2019 (*n* = 698,369, *N* = 186). The black dot represents the mean, and the vertical line represents 1 standard deviation on either side of the dot.

The variation between Twitterers in the production of tweets is huge, ranging from 41 to 40,887 tweets per person. The 186 Twitterers wrote on average 3,755 tweets (sd = 5,506). Figure 2 shows the distribution of the number of tweets per Twitterer. This unequal distribution of tweets over speakers might have an impact on the distribution of our variable too, see below. But language variationists usually deal with this problem by taking per individual a sample of the variable.

**Figure 3:**
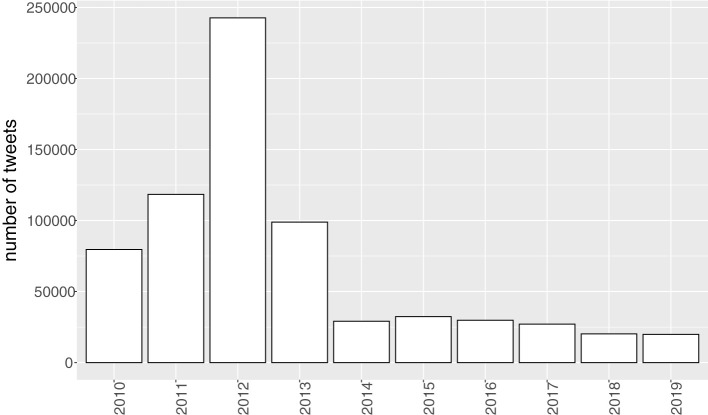
Distribution of the number of tweets per year (*n* = 698,369, *N* = 186).

Figure 3 gives the frequency distribution of the tweets in the past decade. 77.3% of the tweets were retrieved between 2010 and 2013, with a peak in 2012. From 2014 onwards the number of tweets remains stable. It should be noted that this frequency distribution is biased by our retrieval method. We only follow Twitterers that are part of the Twidentity data set, which were active in 2013 and 2014. People who started using Twitter later were not included. Twenty Twitterers posted at least 20 tweets per year. So, the frequency distribution presents the number of tweets in this panel study, it is not a representative frequency distribution of all tweets in this decade.

**Figure 4:**
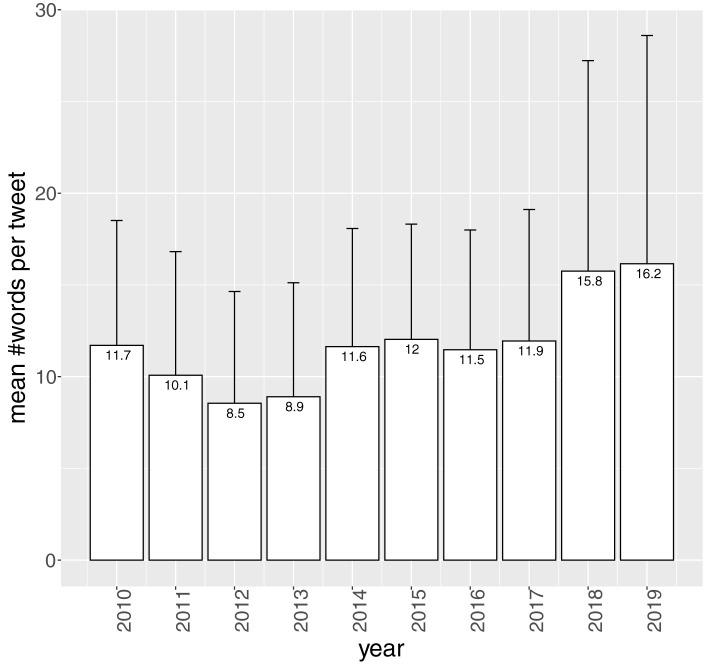
Mean number of words per tweet (averaged over Twitterers), split up by year (*n* = 698,369, *N* = 186).

Figure 4 illustrates the mean number of words per tweet (averaged over Twitterers), split up by year. The mean number of words per tweet remains quite constant over the years. Twitter doubles the number of characters from 140 to 280 in 2017. As becomes clear from Figure 4, this has a moderate impact on the length of tweets. The tweets posted in 2018 and 2019 are a couple of words longer, however, they also show more variance in number of words. This is in line with findings of Glicoric et al. (2020) on number of characters of tweets. Before the switch to 280 characters, 9% of English tweets were exactly the maximum 140 characters long. After the switch still a substantial number of tweets reached the new maximum of 280 characters. They also demonstrated a significant but moderate increase in number of characters, also across languages.

## Selecting Tweets With Frisian Relative Pronouns

### Data Cleaning and Coding

Once the data was retrieved, we first wanted to get more insight in the characteristics of the Twitterers. We tried to detect birth year and gender of the 186 individual Twitterers in the data set (698,369 tweets) by extensive searches on the internet and other public resources. For 82.5% of them, birth year could be retrieved. For the remaining 17.5%, the birth year was estimated based on the user's profile picture and additional public information on Facebook or LinkedIn. One of the Twitterers was an outlier, being born in 1929, since the second oldest person was born in 1945, so his tweets were discarded from the corpus.

Next, we automatically selected all tweets that contained one or more words that were similarly written as one of the target variables (see Table 2 for all possible variants of the target variables). Note that some of these variants are also Dutch words, so this data set comprised Frisian and Dutch tweets, and tweets of which the language was undetermined. As Table 3 shows, we also selected tweets in which the target variables had a suffix—*st* to the pronoun. This is the inflection marker for second person singular that is used when the relative clause has a second person singular subject, e.g., *de auto dêr'tst yn rydst* ‘the car which-you drive’. The (t) before the –*st* suffix is usually not pronounced (Hoekstra, 1985). The automatic selection resulted in a subset of 100,365 tweets from 185 Twitter accounts.

**Table 2:** All variants of the target variables (*dy't*), (*dêr't*), (*wêr't*), and (*wa't*).

**Variable**	**Variants**
(*dy't*)	*dy't, dy'tst, die't, die'tst dy, dy'st, die, die'st*
(*dêr't*)	*dêr't, dêr'tst, der't, der'tst dêr, dêr'st, der, der'st*
(*wêr't*)	*wêr't, wêr'tst, wer't, wer'tst wêr, wêr'st, wer, wer'st*
(*wa't*)	*wa't, wa'tst, wie't, wie'tst wa, wa'st, wie, wie'st*

**Table 3:** Results for CLD3 and textcat (*n* = 698,369).

**CLD3**	**textcat**	**Number of tweets**	**%**	**Language**
Frisian	Frisian	147,585	21.1	Frisian
Frisian	?	23,141	3.3	Frisian
?	Frisian	59,357	8.5	Frisian
Frisian	Dutch	4,530	0.6	Frisian
Dutch	Frisian	3,028	0.4	Frisian
Dutch	Dutch	141,551	20.3	Dutch
Dutch	?	18,780	2.7	Undetermined
?	Dutch	104,870	15.0	Undetermined
?	?	195,527	28.0	Undetermined

*‘?’ means that the language detected was neither Frisian nor Dutch or that the detector was not able to determine the language*.

The variants without /t/ could also be other parts of speech, e.g., the adverb *dêr*, the verb *die* (first and third person singular past tense of *dwaan* ‘to do’), the verb *wie* (first and third person singular past tense of *wêze* ‘to be’), etc. As there was no POS-tagger for Frisian at the time of research, as for most low-resourced languages, the 100,395 tweets with possible realizations of the variables had to be checked manually by the first author who is a native speaker of Frisian. This resulted in a data set with tweets that had at least one of the relative pronouns comprised 5,500 tweets and 5,688 tokens of 159 Twitterers. During the analysis, we saw unexpected variants of the target variables where instead of <'*t*> the adverb *as, at*, ^*^os or ^*^ot was used, i.e., *wer as*, or *wer os*. Tweets with these variants were discarded. Consequently, the final data set contains 5,395 tweets and 5,559 tokens of 159 Twitter accounts.

Finally, the Twitterers were split into two groups based upon the spelling used in their tweets: a spelling following grammatical rules of Frisian and a phonetically oriented one. As mentioned, only 18% of the population of Fryslân claims to be able to write (well) in Frisian (Klinkenberg et al., 2018). Therefore, the spelling habits of the Twitterers might be a factor in the use of t-full or t-less forms. When the spelling was phonetic and/or according to Dutch spelling rules or with many Dutch interferences, the spelling was considered as phonetically oriented spelling. Otherwise, the spelling was coded as standard.

### Automatic Detection of Frisian Tweets

There are three main types of tweets:

1. tweets written in Frisian following Frisian spelling rules.
2. tweets written in Frisian following Dutch spelling rules.
3. tweets written in Dutch following Dutch spelling rules.

Considering the second type, many Twitterers do not use typical Frisian characters such a <û>, <ú> or <y>, but use Dutch <oe>, <uu> or <i> instead. This type of tweets may easily be classified as Dutch by the language detectors. We wanted to see to what extent it was possible to detect automatically the Frisian and Dutch tweets. To this end we used the two language detectors that are available in the R programming language: the function textcat from the textcat package (Hornik et al., 2013), and the function detect_language from the cld3 package (Ooms, 2020). We used the following procedure:

- If one of the detectors classifies a tweet as Frisian, the tweet is coded as Frisian.
- If both detectors classify a tweet as Dutch, the tweet is coded as Dutch.
- In all other cases the language remained undetermined.

The function textcat provides an implementation of the Cavnar and Trenkle (1994) approach to text categorization based on character *n*-gram frequencies. This approach uses two steps. First, training corpora are collected for a set of languages. Each corpus includes texts all written in the same language. For each corpus the frequency distribution of all *n*-grams (*n* = 1.5) found in the texts are computed. Then the *n*-grams are sorted from the most to the least frequent. The *k* most frequent ones are retained and represent a language profile.

In the second step the language of a given text document is identified. A profile is computed, using the same procedure as for the training corpora. Then the text document is classified according to the language of the language profile with the smallest distance to the text document profile. Cavnar and Trenkle (1994) suggest the so-called “out-of-place” distance measure. When measuring the distance between the text document profile and a language profile, for each *n*-gram in the text document profile, we find its counterpart in the language profile and calculate how far out of place it is. For example, if an *n*-gram ranks the second in the text document profile, and the nineth in the language profile, the out of place is seven. If an *n*-gram is not in the category profile, it takes some maximum out-of-place value. The distance between the two profiles is the sum of all of the out-of-place values for all *n*-grams.

The function detect_language is a wrapper for Google's Compact Language Detector 3 (CLD3). CLD3 is a neural network model for language identification. Character *n*-grams are extracted from the input text and the fraction of times each of them appears is computed. For example, ‘banana’ has unigrams ‘b’, ‘a,’ and ‘n’ with fractions 1/6, 3/6, and 2/6, bigrams ‘ba’, ‘na,’ and ‘an’ with fractions 1/5, 2/5, and 2/5, and trigrams ‘ban’, ‘ana,’ and ‘nan’ with fractions 1/4, 2/4, and 1/4. This information is passed to a trained neural network model which subsequently predicts the language. See also: https://github.com/google/cld3#readme

The results for the two language detectors are presented in Table 3. In total, of the almost 700,000 tweets in the corpus, the two language detectors agreed on 69.4% of the tweets as being Frisian (21.1%), Dutch (20.3%), or undetermined (28%). One-third of the total was classified as Frisian by at least one of the detectors. This shows that combining the two language detectors resulted in a high number of Frisian tweets that would otherwise have been discarded. The language detectors disagreed on almost a third of the tweets (30.6%). This might be explained by the close relation between the two languages, and the many code switches and phonetic spelling that were used by the Twitterers.

As mentioned in Section Data cleaning and Coding we found 5,559 tokens in the manual coding. Most tokens (5,314) came from tweets that were detected as Frisian by one or both language detectors. Additionally, the category that was detected as “undetermined” contained a significant number of tokens, i.e., 238 as well. In contrast, the 13,932 tweets that were classified as Dutch by both language detectors had seven tokens from seven tweets (0.05%). In other words, in future analyses it is beneficial to combine the two language detectors and perform a manual check on the Frisian and undetermined tweets and discard the Dutch ones. One would then only miss a small number of tokens and gain a lot of time.

### The Final Set of Tweets With at Least One of the Target Variables (5,395 Tweets, 5,559 Tokens, 159 Twitterers)

The analysis of linguistic variables is often hampered by the unequal distribution of the variable over linguistic contexts or speakers (the frequency problem), the entanglement of linguistic factors resulting in (in)frequent combinations of these factors (the co-occurrence problem) and the existence of (groups of) speakers showing linguistically different patterns of variation (the interaction problem) (Van de Velde and van Hout, 2000).

Table 4 presents the frequency distribution of the variants of the four target variables (*dy't*), (*dêr't*), (*wêr't*) and (*wa't*). The tokens are unequally distributed over the target variables. More than two-thirds of the tokens, i.e., 3,807 (68.4%), are variants of (*dy't*), and the remaining part consists of variants of the other three target variables (co-occurence problem). When looking at the (*dy't*)-variable in more detail we see several variants of the t-full forms, i.e., *dy't*, ^*^die't, dy'tst, and ^*^die'tst. In total we see 3,337 realizations of these t-full forms of *dy't*), which comes down to 87.7%, whereas 470 realizations (12.3%) are t-less variants of (*dy't*). Although most realizations of (*dy't*) are t-full, the variant where the Dutch spelling (^*^die) is used and the variants that have inflection of second person singular (^*^dy(')st and ^*^die(')st) mostly have a t-less form.

**Table 4:** Distribution of Frisian relative pronouns (*dy't*), (*dêr't*), (*wêr't*), and (*wa't*) in the final data set (*n* = 5,559, distributed over 159 Twitterers).

**Target variable**	**t-full forms**	***n***	**t-less forms**	***n***
*(dy't)*	*dy't*	3278	^*^dy	136
	^*^die't	55	^*^die	286
	*dy'tst*	4	^*^dy(')st	24
	^*^die'tst	0	^*^die(')st	24
(d^*^r'*t)*	*dêr't*	408	*dêr*	0
	^*^der't	23	^*^der	0
	*dêr'tst*	2	^*^dêr(')st	15
	^*^der'tst	0	^*^der(')st	1
*(wêr*'*t)*	*wêr't*	521	^*^wêr	78
	^*^wer't	52	^*^wer	98
	*wêr'tst*	1	^*^wêr(')st	21
	^*^wer'tst	0	^*^wer(')st	30
*(wa't)*	*wa't*	334	^*^wa	139
	^*^wie't	1	^*^wie	19
	*wa'tst*	3	^*^wa(')st	4
	^*^wie'tst	0	^*^wie(')st	2
**Total**		**4682**		**877**

* *Ungrammatical variant*.

As for the second most frequent target variable (*wêr't*) (*n* = 801; 14.4%), we see in Table 4 that most realizations are t-full forms, but it seems that the variants without accent, ^*^wer, and with the suffix of second person singular –*st*, are more frequently realized as t-less forms. The variable (*wa't*) (*n* = 502; 9.0%) shows a similar pattern as (*dy't*): most realizations are t-full forms, but the variants where the Dutch spelling (^*^wie) is used and the variants inflection of second person singular (^*^wa(')st and ^*^wie(')st) mostly have a t-less form. The variable (*dêr't*) has the lowest frequency (*n* = 449; 8.1%). Note also that (*dêr't*) is always used with t-full forms, except when it is inflected with the second person singular suffix. In that case, the Twitterers mostly use a t-less form (see Table 4).

On average, there are 35 occurrences of the variable per Twitterer. Table 5 presents the distribution of the number of tokens, and Twitterers, split up for gender, birth year (per decade), writing style (phonetic or standard Frisian). There are no differences between men and women. The tokens show up most frequently in the tweets of Twitterers born between 1951 and 1970. In tweets of Twitterers born after 1980 the frequency of the number of tokens is much lower. In contrast, the younger Twitterers are overrepresented in this data set compared to the older Twitterers. This is in line with findings from Blank (2016) that Twitterers are generally younger than the offline population. The unequal distribution of the variable over the birth cohorts might hamper a robust analysis of this data set from the perspective of language change. 87.5% of the tokens showed up in tweets in standard Frisian orthography, only 12.5% in tweets with phonetic spelling. This supports previous observations that in CMC Twitter mainly shares characteristics with traditional writing styles (Verheijen, 2018).

**Table 5:** Characteristics of the Twitterers: gender, birth year (per decade) and spelling style (standard or phonetic Frisian) per number of tokens (*n* = 5,559) and Twitterers (*N* = 159).

**Variable**	**Categories**	***n* tokens**	***N* twitterers**
Gender	Male	2,779	81
	Female	2,780	78
Birth year	1941–1950	870	9
	1951–1960	1,431	22
	1961–1970	1,484	24
	1971–1980	890	21
	1981–1990	388	29
	1991–2000	496	54
Spelling style	Standard Frisian	4,863	83
	Phonetic Frisian	696	76

**Figure 5:**
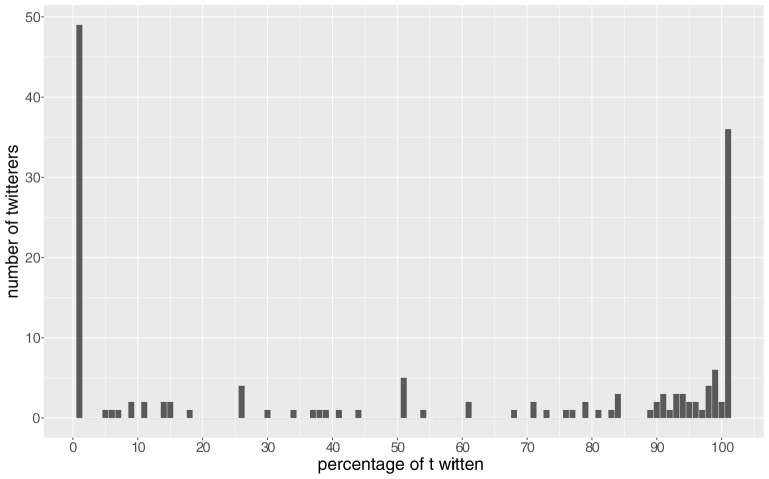
Distribution of number of Twitterers per percentage of t-full forms used (*N* = 159).

**Figure 6:**
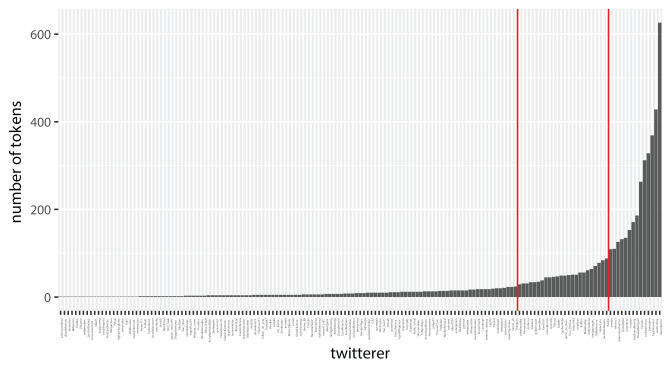
Number of tokens per Twitterer (*N* = 159).

Figure 5 shows the number of Twitterers distributed over the percentage of using t-full forms in their tweets. Forty-nine Twitterers (30.8%) never use the t-full forms and 36 Twitterers (22.6%) always use the t-full forms. Seventy-four Twitterers (46.5%) vary in their use of t-full and t-less forms. Figure 6 illustrates in more detail the unequal frequency distribution of the tokens. It should also be noted that 21 Twitterers use the target variable only once in their tweets posted in the decade 2010–2019. Such an unequal distribution of tokens (frequency problem) might hamper a solid analysis of the factors influencing the use of t-full variants. It is likely that the Twitterers with many tokens are the most-skilled writers, with solid knowledge of Frisian normative rules and standard orthography (interaction problem).

When ranking the final set of tweets with at least one of the target variables by birth year (see Table A that is uploaded as Supplementary Material), we see that without exception the Twitterers born after 1988 stop tweeting after 2014. The fact that the older Twitterers produce most of the tokens and the younger Twitterers leave Twitter after 2014, which is a problem for the analysis of the interaction of time and age in our panel study.

In an attempt to cope with the above problems related to the distribution of the tokens, the Twitterers were split in three groups. A small group of Twitterers (*n* = 14), FreqvarH, realizes two-thirds of the total amount of tokens in the data set (see Table 6 and Figure 6). Most of the Twitterers (*n* = 118) produce a relatively low number of tokens of the variable (FreqvarL, range 1–21). We also created an intermediate group, FreqvarM (*n* = 27, range = 23–88). In the results section we will present separate analyses for these groups.

**Table 6:** Distribution of number of Twitterers (*N* = 159) and tokens (*n* = 5,559), split up in three frequency groups.

	**Range per Twitterer**	***n* tokens**	***N* Twitterers**
FreqvarL	1–21	818	118
FreqvarM	23–88	1,293	27
FreqvarH	106–626	3,448	14

### Analysis of Tweets of Final Data Set

The data were analyzed using a mixed-effects logistic regression model in R (The R Foundation for Statistical Computing, http://CRAN.R-project.org/) by applying the glmer function in the lme4 package (Bates et al., 2015). This function uses a combination of Nelder-Mead and bobyqa as optimizer. The model did not converge when it included the variable “pronoun.” Therefore, we used the optimizer nlminb from the R package optimx, which solved the conversion issues. The code of nlminb() was written by David Gay at Bell Labs and part of the Fortran library (Fox et al., 1978).

The dependent variable was (t) with 0 indicating a t-less form and 1 indicating a t-full form. The analysis started with an initial model that included the following fixed factors: birth year and gender of the Twitterers, the year in which the tweet was posted, the spelling style, the pronoun, the number of words used in the tweet, the number of tokens per Twitterer, the percentage of Frisian used in the tweets, and the presence of the suffix –*st*. The percentage of Frisian per Twitterer is calculated as the number of Frisian tweets divided by the total number of tweets multiplied by 100. The number of (Frisian) tweets is calculated on the basis of the full data set, i.e., the set which contains all tweets regardless of the presence of any of the target words. A tweet is considered Frisian when it is detected as Frisian by any of the two language detectors that we used (see Section Automatic Detection of Frisian Tweets).

The Twitterers were included as a random factor, to control for individual differences. The variable pronoun was included as random slope. Starting with the initial model, backward analysis was acquired to obtain the best model that had a significant improvement of the Akaike Information Criterion (AIC). We followed this procedure for each of the three groups of Twitterers (low, moderate and high frequency of the variable) from Table 6. The optimal models from these analyses are presented in the next section.

## Results

We present separate analyses for the groups with low, moderate, and high frequency of the variable.

The optimal model for the FreqvarL-Twitterers, i.e., Twitterers who use a small number of tokens (1 up to 21), is presented in Table 7. Younger Twitterers substitute the Frisian relative pronouns significantly more with the t-less forms of relative pronouns, compared to older Twitterers. Further, longer tweets (in terms of number of words), have significantly more t-less forms. Also, phonetic spelling is an important factor. Twitterers who tweet in phonetic Frisian use significantly more t-less forms. Pairwise comparisons for pronouns show that significantly more t-less forms are found for *dy't* compared to *wêr't* (z = 2.69, *p* < 0.05).

**Table 7:** Optimal model for substitution of Frisian relative pronouns *dy't, dêr't, wêr't*, and *wa't* in Twitter data for FreqvarL, Twitterers who infrequently produced the variable (range = 1–21) in their tweets (*n* = 818, *N* = 118).

	**Estimate**	**S.E**.	***Z* value**	**Pr (>|z|)**
Intercept	3.65	1.18	3.08	*p* < 0.001
Birth year	−1.60	0.40	−3.96	*p* < 0.00
#words	0.31	0.15	2.06	*p* < 0.05
Phonetic spelling	−2.98	0.80	−3.73	*p* < 0.001
*(dy't)*	−0.36	1.13	−0.32	n.s.
*(wa't)*	−1.38	1.17	−1.18	n.s.
*(wêr't)*	−1.29	1.16	−1.11	n.s.

Table 8 presents the optimal model for FreqvarM-Twitterers who moderately use the variable (23 up to 88) in their tweets. Within this model the suffix –*st* is also included as a random slope. The model shows that within this group the year of posting is a significant factor. The more recent the posting, the more t-full forms were used. Additionally, the Twitterers using phonetic spelling used significantly more t-less forms compared to those using standard Frisian spelling. The addition of the suffix –*st*, when the subject in the relative clause is second person singular, also triggers the use of t-less relative pronouns. Pairwise comparisons for pronouns show that significantly more t-less forms are found for *dy't* and *wa't* (z = 5.31, *p* < 0.001) and *wêr't* (z = 7.48, *p* < 0.001). Variables such as birth year, gender, the number of words in a tweet or the percentage Frisian used in the tweets were not included in this model.

**Table 8:** Optimal model for substitution of Frisian relative pronouns *dy't, dêr't, wêr't*, and *wa't* in Twitter data for FreqvarM, Twitterers who moderately produced the variable (range = 23–88) in their tweets) (*n* = 1,293, *N* = 27).

	**Estimate**	**S.E**.	***Z* value**	**Pr (>|z|)**
Intercept	6.74	2.68	2.52	*p* < 0.05
Year of posting	0.37	0.17	2.07	*p* < 0.05
Phonetic spelling	−6.15	0.86	−7.14	*p* < 0.001
Suffix -*st*	−16.94	5.00	−3.39	*p* < 0.001
*(dy't)*	−2.30	2.65	−0.87	n.s.
*(wa't)*	−4.46	2.67	−1.67	n.s.
*(wêr't)*	−5.02	2.66	−1.89	n.s.

Table 9 presents the optimal model for the FreqvarH-Twitterers, i.e., Twitterers with a high frequency of the variable (range: 106–626) in their tweets. A fixed effect is found for the variable count. This variable represents the number of tweets that a Twitterer posted between January 1, 2010 and December 31, 2019. It means that Twitterers who posted more tweets used more t-full forms. Additionally, when the suffix –*st* is cliticized to the relative pronoun, the pronoun itself shows up more frequently as a t-less form. The table further shows an effect for pronoun. Pairwise comparisons show that significantly more t-less forms are found for *dy't* and *wa't* (z = 6.85, *p* < 0.001) and *wêr't* (z = 4.50, *p* < 0.01), and more t-full forms for *dêr't* compared to *wa't* (z = 3.70, *p* < 0.01) and *wêr't* (z = 2.93, *p* < 0.05). The variables birth year, gender, year of posting, number of words of a tweet, spelling style, or the percentage of Frisian in the tweet do not significantly contribute to the optimal model.

**Table 9:** Optimal model for substitution of Frisian relative pronouns *dy't, dêr't, wêr't*, and *wa't* in Twitter data for FreqvarH, i.e., Twitterers who frequently produced the variable (range = 106–626) in their tweets) (*n* = 3,448, *N* = 14).

	**Estimate**	**S.E**.	***Z* value**	**Pr (>|z|)**
Intercept	6.51	1.08	6.01	*p* < 0.001
Count	1.21	0.50	2.42	*p* < 0.05
Suffix -*st*	−8.39	0.94	2.06	*p* < 0.05
*(dy't)*	−1.78	1.01	−1.76	n.s.
*(wa't)*	−3.81	1.03	−3.70	*p* < 0.001
*(wêr't)*	−3.00	1.02	−2.93	*p* < 0.01

## Discussion and Conclusion

The current study investigated a change in progress in Frisian based on Twitter data: the substitution of t-full relative pronouns *dy't, dêr't, wêr't*, and *wa't* with their t-less counterparts. The aim of the study was threefold. First, we wanted to explore the issues in gathering a Twitter corpus of a low-resource language such as Frisian. Second, we wanted to get more insight in the validity of Twitter data for the study of language change in progress. Third, we tried to enhance our insight in a vigorous change in progress.

### Collecting Data

The collection of Frisian Twitter data turned out to be a complex process. Multiple requests to get permission to retrieve tweets from Twitter for linguistic research did not result in an answer from Twitter. Consequently, we used the GetOldTweets3-script to retrieve the tweets of a fixed set of individual Twitter accounts of Frisian Twitterers that were previously identified and selected in another project (Jongbloed-Faber et al., 2017). A corpus of almost 700,000 predominantly Frisian and Dutch tweets posted in the decade 2010–2019 was collected. After automatically selecting the possible realizations of the variables and a check analysis, we ended up with the final data set of 5,395 Frisian tweets with one or more realizations of the variables, which is a fraction (0.8%) of the tweets from the entire Twitter data set. Although being recognized as the second official language in the province, Frisian is not omnipresent in the written domain and only a small proportion of the 450,000 speakers of Frisian write Frisian. Hence, data sets and corpora of such languages are much smaller than the ones of majority or medium-sized languages. Furthermore, many Twitterers of a minority language are bilingual and tweet in the majority language (as well). Evidence from a previous study (Jongbloed-Faber et al., 2017) showed that the Twitterers from our data set used (some) Frisian in their tweets, next to Dutch (or occasionally another language). Jongbloed-Faber et al. (2016) also pointed out that 65% of the Frisian teenagers never use Frisian in their tweets. So, the Twitterers of our data set might give a slightly distorted view of the language use of the average Frisian Twitterers who predominantly use Dutch.

### Detection of the Language

The automatic identification of tweets as Frisian or Dutch was not very successful. Almost one-third of the tweets was classified as undetermined and these tweets contained a significant number of tokens of our linguistic variable. Frisian and Dutch are closely related languages, and the Frisian lexicon contains a lot of Dutch loans. The fact that a large part of the Frisian tweets is written in phonetical spelling, heavily influenced by Dutch spelling conventions, make automatic distinction between Frisian and Dutch tweets even more difficult. Furthermore, the corpora behind the language detector textcat are relatively small, which makes such a detector less performant. Like most minority and smaller languages, Frisian is technologically a low-resource language and at the time of our research a POS-tagger for Frisian was not available. A POS-tagger for Frisian would have made it easier to distinguish between *dy* used as t-less relative pronoun or demonstrative pronoun, *wêr* used as t-less relative pronoun or interrogative pronoun, *wer* as relative pronoun or adverb, *wa* as relative pronoun or interrogative pronoun, *wie* as (Dutch) relative pronoun or (Frisian) inflection of *wêze* ‘to be’. Consequently, the 100,365 tweets had to be analyzed manually to distinguish the target variables from other words.

### Analysis and Imbalance of Data

Three of the four relative pronouns in our study had a low frequency in comparison with the fourth one. This co-occurrence problem makes it difficult to study the role of this linguistic factor and might explain why it does not show up as a significant factor in the low and medium frequency groups.

Differences in the quantity of tweets is not a problem, if a comparable sample of tokens per individual can be selected. However, two-thirds of the tokens of our variable are produced by less than one-tenth of the Twitterers in our corpus, and most of the Twitterers produce a low number of tokens in the decade we were able to track their tweets. This unequal distribution of tokens is problematic for a panel study of language change in progress.

The final data set appeared to be even more biased. In our panel study, we observe a strong decrease in the use of the medium. Most tweets are posted between 2011 and 2013. After 2014 there is a rapid decline in the number of tweets. Striking is that all Twitterers in our data set born after 1988, without any exception, stopped posting on Twitter after 2014. This is a problem for panel studies like this one, especially in low resource languages where the amount of data is rather limited. Furthermore, most tokens of our variable are produced by Twitterers from older generations, hampering an analysis of the data set in apparent time, and the interaction of age and period.

### Findings

The shortcomings of the data set did not imply that we could not refine the existing insights in this change in progress, since the data set showed two interesting observations. The first observation concerned the target variable *dêr't*. A previous study on radio speech data showed that the target variable *dêr't* was mostly found in scripted radio speech and almost always in t-full form (Dijkstra et al., 2019). The current study demonstrated that in tweets, the t-full form is always used in *dêr't* (unless this relative pronoun is inflected with second person singular suffix –*st*). This suggests that the relative pronoun *dêr't* is part of written rather than of oral Frisian. A second observation concerns the suffix –*st*. The suffix –*st* seems to trigger the t-less form of all target variables predominantly in tweets from the two most active groups of Twitterers. This might be explained by the observation that the /t/ before the –*st* suffix is usually not pronounced (Hoekstra, 1985). Due to the bias in the data set, we have to be careful in generalizing our findings. They need to be confirmed on the basis of additional analysis of spoken and written corpora.

When studying language change in panel studies one needs to monitor individuals over a period of time. The instability in token production by individuals and the general decline of the medium, especially amongst young Twitterers, make it hard to demonstrate language change in progress in real time. Our analyses were further hampered by the fact that the variables had a low frequency and were unequally distributed over Twitterers. The fact that the language under investigation was a low-resource language, made the search and analysis even more challenging. In conclusion, for low-frequency variables in low-resource languages, Twitter is unlikely to be an appropriate source for quantitative sociolinguistic studies of language change in progress.

## Data Availability Statement

The original contributions presented in the study are included in the article/Supplementary Material, further inquiries can be directed to the corresponding author/s.

## Ethics Statement

Written informed consent was not obtained from the individual(s) for the publication of any potentially identifiable data included in this article as the data were anonymized.

## Author Contributions

JD, WH, LJ-F, and HV all collaborated on the conception of the study. The design was developed by JD, WH, and HV. Data was collected by LJ-F and WH and prepared by WH. Data was manually analyzed by JD. Statistical analyses were performed by WH. A first draft of the paper was written by JD and HV. WH and LJ-F (co-)wrote sections of the paper. JD and HV prepared the final version of the manuscript which was read, revised, and approved by WH and LJ-F. All authors contributed to the article and approved the submitted version.

## Conflict of Interest

The authors declare that the research was conducted in the absence of any commercial or financial relationships that could be construed as a potential conflict of interest.
