# Supplementary material for: Using Twitter Data for the Study of Language Change in Low-Resource Languages. A Panel Study of Relative Pronouns in Frisian
Source: Front Artif Intell. 2021 Apr 15;4:644554. doi: 10.3389/frai.2021.644554 (PMC8083371; doi:10.3389/frai.2021.644554)
Supplement: Supplementary file 3 [file Table_1.docx]

Table A. Number of Twitterers ranked per birth year and year (N=159).

|  | **2010** | **2011** | **2012** | **2013** | **2014** | **2015** | **2016** | **2017** | **2018** | **2019** | **sum** |
| --- | --- | --- | --- | --- | --- | --- | --- | --- | --- | --- | --- |
| 1945 | 1 | 1 | 1 | 1 | - | - | - | 1 | 1 | 1 | 7 |
| 1946 | 2 | 1 | 1 | 2 | - | 2 | 1 | 3 | 2 | 2 | 16 |
| 1947 | - | 1 | - | 1 | 1 | - | - | - | - | - | 3 |
| 1948 | 1 | 1 | 1 | 1 | 1 | 1 | 1 | 1 | 1 | 1 | 10 |
| 1950 | - | 2 | 1 | 1 | 2 | 1 | 1 | 1 | 1 | - | 10 |
| 1952 | - | - | 1 | 1 | - | - | - | - | - | - | 2 |
| 1954 | 3 | 3 | 3 | 3 | 3 | 3 | 3 | 3 | 2 | 2 | 28 |
| 1955 | - | 1 | 1 | 2 | 1 | 1 | 1 | 1 | 1 | - | 9 |
| 1956 | 1 | 4 | 3 | 5 | 3 | 2 | 2 | 3 | 2 | 1 | 26 |
| 1957 | 1 | 2 | 2 | 1 | - | 1 | 1 | 1 | 1 | 1 | 11 |
| 1958 | - | 1 | 1 | 1 | 1 | 2 | 2 | 1 | - | - | 9 |
| 1959 | 1 | 1 | 2 | 3 | 2 | 2 | 1 | 1 | 1 | 1 | 15 |
| 1960 | - | 1 | 1 | 1 | 2 | 1 | - | - | - | - | 6 |
| 1961 | - | 1 | 1 | 3 | 1 | 1 | 2 | 1 | 2 | 1 | 13 |
| 1963 | 1 | 4 | 3 | 4 | 2 | 2 | 1 | 2 | 2 | 3 | 24 |
| 1964 | - | 1 | 2 | 2 | 2 | - | - | - | 1 | 1 | 9 |
| 1965 | 2 | 1 | 2 | 2 | 1 | 1 | 1 | 1 | 1 | 1 | 13 |
| 1966 | - | 1 | 1 | 1 | 1 | - | 1 | 1 | 1 | 1 | 8 |
| 1967 | 2 | 2 | 3 | 3 | 2 | 2 | 2 | 2 | 1 | 2 | 21 |
| 1968 | 2 | 2 | 3 | 3 | 3 | 3 | 2 | 2 | 3 | 1 | 24 |
| 1969 | - | - | - | - | - | - | - | 1 | 1 | 1 | 3 |
| 1970 | - | 1 | 1 | 1 | 1 | - | 1 | 1 | - | - | 6 |
| 1971 | - | 1 | 1 | 1 | 1 | 1 | 1 | 1 | 1 | 1 | 9 |
| 1972 | - | 2 | 3 | 1 | 3 | 1 | 1 | 1 | 1 | 1 | 14 |
| 1973 | - | 2 | 2 | 2 | 1 | 1 | 1 | 1 | 1 | 1 | 12 |
| 1974 | - | - | 1 | 1 | 1 | 2 | 2 | 2 | 1 | - | 10 |
| 1975 | 1 | 1 | 1 | 1 | 1 | 1 | 1 | - | 1 | 1 | 9 |
| 1976 | - | 2 | 4 | 4 | 3 | 3 | 3 | 2 | 2 | 1 | 24 |
| 1977 | - | 1 | 1 | 1 | 1 | 1 | 1 | 1 | - | - | 7 |
| 1978 | 1 | 1 | 2 | 2 | - | 1 | - | - | - | - | 7 |
| 1979 | 1 | 2 | 1 | 2 | 2 | 1 | 1 | 1 | 1 | 1 | 13 |
| 1980 | - | 1 | 2 | 1 | 1 | - | 1 | - | - | - | 6 |
| 1981 | - | 1 | 1 | 2 | - | - | - | - | - | - | 4 |
| 1982 | 1 | 2 | 1 | 3 | 1 | 1 | 2 | 2 | 2 | 2 | 17 |
| 1983 | 1 | 3 | 4 | 1 | 1 | - | 1 | - | - | 1 | 12 |
| 1984 | - | - | - | - | 1 | 1 | 1 | 1 | - | - | 4 |
| 1985 | - | 1 | 2 | 2 | 1 | 2 | 2 | - | - | 1 | 11 |
| 1986 | 1 | 3 | 3 | 2 | 2 | 2 | 1 | - | 2 | - | 16 |
| 1987 | - | 1 | 1 | 1 | 1 | 1 | 1 | - | 1 | 1 | 8 |
| 1988 | - | - | 1 | - | - | - | - | - | - | - | 1 |
| 1989 | - | 1 | 3 | 2 | 1 | - | - | - | - | - | 7 |
| 1990 | 1 | 4 | 4 | - | - | - | - | - | - | - | 9 |
| 1991 | 2 | 2 | 3 | - | - | - | - | - | - | - | 7 |
| 1993 | 2 | 2 | 5 | 4 | 1 | - | - | - | - | - | 14 |
| 1994 | - | 5 | 6 | 4 | - | - | - | - | - | - | 15 |
| 1995 | - | 1 | 5 | 6 | - | - | - | - | - | - | 12 |
| 1996 | - | 5 | 9 | 7 | - | - | - | - | - | - | 21 |
| 1997 | - | 5 | 10 | 9 | 2 | - | - | - | - | - | 26 |
| 1998 | - | 1 | 5 | 4 | - | - | - | - | - | - | 10 |
| 1999 | - | - | - | 2 | - | - | - | - | - | - | 2 |
| 2000 | - | - | 1 | 1 | - | - | - | - | - | - | 2 |
